# Corrigendum to “*Lycium* Berry Polysaccharides Strengthen Gut Microenvironment and Modulate Gut Microbiota of the Mice”

**DOI:** 10.1155/2020/4840656

**Published:** 2020-11-25

**Authors:** Wenrui Xia, Xiaoang Li, Lu Su, Imran Khan, Wai Kit Leong, Lin Yin, Xiqing Bian, Jiyan Su, Guoxin Huang, W. L. Wendy Hsiao

**Affiliations:** ^1^State Key Laboratory of Quality Research in Chinese Medicine, Macau University of Science and Technology, Macau, China; ^2^Research Laboratory, Foshan Women and Children Hospital, Affiliated Foshan Women and Children Hospital of South Medical University, Foshan 528000, China

In the article titled “*Lycium* Berry Polysaccharides Strengthen Gut Microenvironment and Modulate Gut Microbiota of the Mice” [1], the order of authors was incorrect. The correct author list and co-corresponding authors is as above.
